# CogWatch: An open-source platform to monitor physiological indicators for cognitive workload and stress

**DOI:** 10.1016/j.ohx.2024.e00538

**Published:** 2024-05-23

**Authors:** Louis J. Dankovich, Janell S. Joyner, William He, Ahmad Sesay, Monifa Vaughn-Cooke

**Affiliations:** a University of Maryland at College Park, James A. Clark School of Engineering, 8228 Paint Branch Dr, College Park, MD 20742, United States; b Virginia Tech, VT Carilion School of Medicine, 2 Riverside Circle, Roanoke, VA 24016, United States

**Keywords:** Wearable, Sensor, State Detection, Heart rate, Galvanic skin response, And educational tools

## Abstract

Cognitive workload is a measure of the mental resources a user is dedicating to a given task. Low cognitive workload produces boredom and decreased vigilance, which can lead to an increase in response time. Under high cognitive workload the information processing burden of the user increases significantly, thereby compromising the ability to effectively monitor their environment for unexpected stimuli or respond to emergencies.

In cognitive workload and stress monitoring research, sensors are used to measure applicable physiological indicators to infer the state of user. For example, electrocardiography or photoplethysmography are often used to track both the rate at which the heart beats and variability between the individual heart beats. Photoplethysmography and chest straps are also used in studies to track fluctuations in breathing rate. The Galvanic Skin Response is a change in sweat rate (especially on the palms and wrists) and is typically measured by tracking how the resistance of two probes at a fixed distance on the subject's skin changes over time. Finally, fluctuations in Skin Temperature are typically tracked with thermocouples or infrared light (IR) measuring systems in these experiments. While consumer options such a smartwatches for health tracking often have the integrated ability to perform photoplethysmography, they typically perform significant processing on the data which is not transparent to the user and often have a granularity of data that is far too low to be useful for research purposes. It is possible to purchase sensor boards that can be added to Arduino systems, however, these systems generally are very large and obtrusive. Additionally, at the high end of the spectrum there are medical tools used to track these physiological signals, but they are often very expensive and require specific software to be licensed for communication. In this paper, an open-source solution to create a physiological tracker with a wristwatch form factor is presented and validated, using conventional off-the-shelf components. The proposed tool is intended to be applied as a cost-effective solution for research and educational settings.

## Graphical abstract

**Figure:**
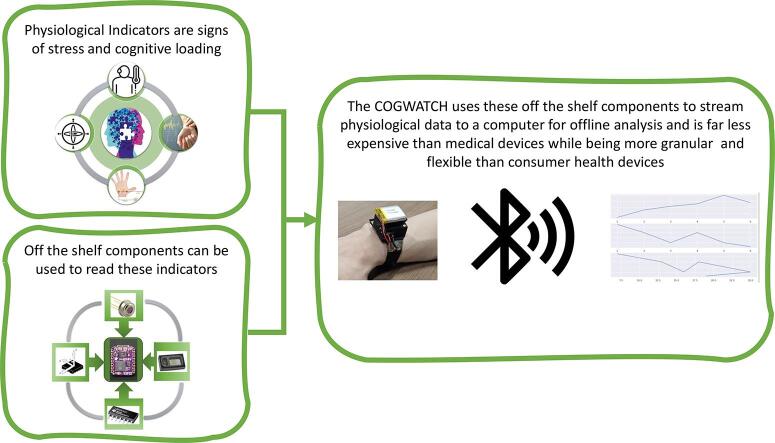

## 1 Specifications table

**Table:** 

Hardware name	CogWatch Open-source Physiological Monitoring System
Subject area	Educational tools and open-source alternatives to existing infrastructure
Hardware type	Wearable Sensors for Physiological Testing
Closest commercial analog	*FitBit, Zephyr, AppleWatch*
Open source license	*CERN-OHL-S*
Cost of hardware	*∼$100*
Source file repository	http://doi.org/10.17605/OSF.IO/SZUYW

## 2 Hardware in context

Physiological indicators are often monitored in research to measure pain, stress, cognitive workload, the onset of disease, and responses to physical activity [1], [2], [3], [4], [5], [6], [7]. These conditions cause activations of the autonomic nervous system (ANS) and the results can be observed by monitoring: heart rate (HR), heart rate variability (HRV), blood volume pressure (BVP), skin temperature fluctuations (STK), galvanic skin responses (GSR), photoplethysmography (PPG), and respiration rate (RR) [3], [7] (Table 1)

**Table 1:** Abbreviations.

Abbreviation	Term
SKT	Skin Temperature Fluctuation
GSR	Galvanic Skin Response
ANS	Autonomic Nervous System
BVP	Blood Volume Pressure
HR	Heart Rate
HRV	Heart Rate Variability
RR	Respiration Rate
BLE	Bluetooth Low Energy
IC	Integrated Circuit
PPG	Photoplethysmography
ECG	Electrocardiography
API	Application Interface
IDE	Integrated Development Environment
COTS	Conventional Off the Shelf Components
ACC	Accelerometer
DOF	Degree of Freedom
IMU	Inertial Motion Unit
Wi-Fi	Wireless Network Protocol

Significant recent attention has been given to wrist worn solutions due to their unobtrusive nature and convenience [8], [9], [10], [11], [12], [13]. The market for wearable health sensors is expected to reach $4,645,160 million by 2026 [14], and currently includes a wide variety of commercial wrist-based physiological sensor solutions (e.g., FitBit, Garmin, Apple Watch, Galaxy Gear) that monitor vitals including HR, RR, SKT, and in some cases GSR. Most commercial devices suffer from one or more of the following issues, which limit feasibility in research and educational settings:

a) Low Speed: In many consumer devices the granularity of data is inadequate. For GSR data typical sampling rates range from 1 to 4 HZ [15]. To reconstruct HRV from PPG sensors the minimum recommended sampling rates found in papers ranged from 100 Hz to 125 Hz [16], [17]. The maximum frequency of sampling provided by Fitbit via their API is 1 Hz [18], and the AppleWatch provides a lower 0.2 Hz frequency [19]. While potentially useful for fitness, this recording rate is far too low to evaluate stress and cognitive workload.
b) Lack of Data Transparency: Raw sensor data is not generally available from fitness watch APIs, and the processes used to generate final values for physiological indicators such as HR and RR from the sensors are not typically exposed or explained [18], [19].
c) Lack of Data: Many devices either do not have or do not expose an adequate number and variety of sensors to be useful. For example, Apple Watch and Fitbit do not include GSR sensors.
d) Cost: Often the cost of devices marketed for research use can be thousands of dollars, which may be prohibitive for use in educational settings and laboratories.
e) Proprietary Software: Software for devices is often complex and poorly documented.

In recent years a significant number of open source software tools have been designed to process the data from physiological sensors, which can help to address the challenges of commercial electronics sensor software packages [20], [21], [22], [23]. However, the hardware side of physiological monitoring does not have nearly as many open source options.

In this work, the Cogwatch (an inexpensive and empirically validated physiological sensor) is proposed to lower financial barriers to research. The system was constructed from easily accessible and programmable conventional-off-the-shelf components (COTS) and does not require significant programing or electronics expertise; making it possible to build in research and educational settings.

## 3 Hardware description

A limited number of open source systems are cited in the literature for the purpose of monitoring cognitive workload and stress. These systems all have barriers to adoption in educational settings and laboratories such as form factor, connectivity, or programming interfaces. Many of these devices were designed by groups with an in-depth research knowledge of electrical engineering, circuit design and building, and microcontroller programming. While these devices present promising solutions for physiological sensing, many engineering and biological research groups will not have the skills to build these systems in-house or the excess funds to subcontract to a professional. Many existing options are limited to Bluetooth Low Energy (BLE) as a wireless communication option [24], [25], [26], [27]. BLE has become more common, is great for extending battery life, and works well with cell phones. However, the learning curve to implement BLE in microcontroller and application environments is non-trivial.

Table 2 contains details on selected open-source options for cognitive workload and stress monitoring found in literature. This list was developed by reviewing recent publications focused on the design and build of wearable cognitive workload and stress monitoring devices. In addition, commercially available options are included in the table for comparison.. The Zephyr 3.0 is included as an example of a commercial device built specifically for tracking bio-signals, which has been used in cognitive workload and sports research. It tracks HR and HRV via ECG rather than PPG and uses onboard sensors to calculate core body temperature (this is different from SKT and generally not applicable for research in this area). It can also communicate wirelessly but requires proprietary software and a specialized hub. The Table 2 Micro Controller column indicates the specific microcontroller used in the system (if the data was available). The Wrist Wearable column indicates whether a device can be worn on the wrist. IDE Access indicates whether the source files can be modified and uploaded via standard Arduino tools, which have a lower learning curve than directly programming microcontrollers. The Raw Data column indicates whether raw data from the device is available. The Wireless column notes the form of wireless communication available (if any). PPG, GSR, SKT, ACC (accelerometer), and ECG columns indicate if the given monitoring sensor is present.

**Table 2:** Selected biosensor options.

Item	Micro Controller	Wrist Wearable	IDE Access	Raw Data	Communication Options	PPG	GSR	SKT	ACC
Mohemaddi et al [24]	NRF52832	No	No	No	BLE	Yes	Yes	No	No
Biotracker[25]	STM32L1	No	No	No	BLE	Yes	No	Yes	Yes
Wang[26]	NRF52832	Yes	Yes	Yes	BLE	Yes	No	No	No
Robust Driver[27]	MSP430G2553	Yes	No	Yes	BLE	Yes	Yes	Yes	Yes
Apple Watch	UNK	Yes	No	No	BLE,WiFi	Yes	No	Yes	Yes
FitBit	UNK	Yes	No	No	BLE,WiFi	Yes	No	Yes	Yes
Zephyr 3	UNK	No	No	No	Communication hub	No	No	No	Yes
Cogwatch	ESP32	Yes	Yes	Yes	Bluetooth, WiFi, USB, BLE available	Yes	Yes	Yes	Yes

The proposed Cogwatch device focusses on a design that does not require specialized techniques to build or program, and which exposes raw data from a variety of physiologically meaningful sensors. Key features include the following notable elements:

• Unlike offering such as the FitBit and AppleWatch, the Cogwatch provides raw data from PPG, GSR, and SKT sensors at an adequate rate to reconstruct physiologically meaningful signals for stress and cognitive workload.
• Unlike many offerings in contemporary research, the Cogwatch is built entirely from conventional off-the-shelf (COTS) components, eliminating specialized equipment as a barrier to construction. These parts are acquired from stable product lines and are expected to remain in production for the foreseeable future.
• The Cogwatch is completely software agnostic. Rather than locking the user into a licensed proprietary software package, the sensor data is streamed in a serial format allowing users to select the tools best suited to their project and software skills.
• The Cogwatch is built on the ESP32 microcontroller which offers several advantages to research groups electing to user this platform:
o A wide variety of communication options are available on the ESP32 including USB serial, Bluetooth Classic, BLE, and WiFi. The provided firmware exposes the USB and Bluetooth Classic ports for interface control and data streaming.
o The ESP32 microcontroller can be programmed entirely using the Arduino IDE or Micropython environments. This lowers the skill barrier to implementing custom features by modifying firmware.

The key design criteria considered include:

• Simplified Assembly: The ability to assemble by hand without specialized equipment was a key consideration. While access to pick and place equipment and a reflow oven are useful, we validated a unit hand assembled by authors with soldering paste, a soldering iron, a magnifying glass, and toaster oven to handle reflow on the surface mounted chips.
• Minimal Footprint: Efforts were taken to ensure that the footprint on the body was minimized.
• Flexibility: While envisioned as a wristwatch form factor with small changes, the Cogwatch can be worn around the ankle, neck, or on a chest strap to obtain data from different locations depending on a project’s needs.
• Parts Availability: This design was implemented shortly after the COVID-19 pandemic during a period where supply chains were disrupted. The components selected were mature product lines which we anticipate being widely available in the foreseeable future.
• Programming options: An ESP32 based microcontroller was selected for this system because it allows for a variety of programming paradigms including Arduino IDE, MicroPython, and directly uploading microcontroller code via the ESP32 toolchain environment.
• Communication options: An ESP32 was selected as a microcontroller because it can transmit over WiFi, Bluetooth, BLE, and serial USB. This allows the end user to select the communication mode best suited to the project’s needs.
• Flexible Sensor Configuration: While our current research is focused on cognitive workload in stationary subjects, the inclusion of an Accelerometer and Magnetic sensor in the design gives the option of looking at patterns of motion and correcting motion artifacts from other sensors in studies involving dynamic motion. It also potentially allows the use off the Cogwatch in motion and health studies which are not directly related to cognitive workload or stress.

**Fig. 1:**
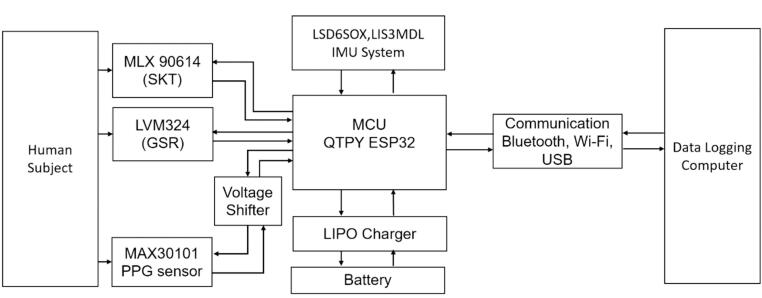
Block Diagram of Cogwatch Components.

A high-level block diagram of the system can be seen in Fig. 1.

An Adafruit QTPY ESP32 dev board was selected as the central microcontroller for this system. The QTPY can communicate wirelessly over Bluetooth Classic, BLE, and Wi-Fi [28]. The Wi-Fi communication allows for network time to be obtained from Universal Time Protocol (UTP) servers if desired. Physiological sensors were selected to measure the indicators typically used in stress and cognitive workload models with ease of implementation, cost, and stability of the product line as key metrics to select sensors.

HR and HRV are typically measured with either ECG or PPG sensors. ECG generally requires multiple wire connected electrodes to be attached to the body via conductive adhesive. These electrodes can be broadly spaced. In addition, sweat can degrade signals over time and the wires can become an impediment in daily wear and physical activities. PPG can be implemented on a variety of body sites including earlobes, finger tips, wrist, and neck; it eliminates wires by using an optical method to predict HR based on blood oxygenation [16], [29]. Historically the clinical gold standard for HR and HRV has been ECG [11], [17], but many studies have found that PPG derived HRV values are acceptably close to those of ECG [16], [29], [30]. Both PPG and ECG have been shown to be useful in deriving RR and BVP, with PPG being slightly better for BVP [31], [32], [33]. It has also been shown that PPG can work well at lower recording rates than ECG requires [34], [35], [36]. Given the open-source nature of this project HeartPy was used for preliminary analysis of HR and to extract RR data [22]. Further analysis was performed in Neurokit2 which has superior tools for analyzing and displaying data pertaining to RR variability (RRV), HRV and GSR [37]. In cognitive workload and stress research, the HRV of interest is in the range 50 ms. This translates to a 20 Hz frequency for the signal of interest, which sampling theory indicates a minimum sampling rate of 40 Hz to capture [38].

To monitor HR and RR, the Maxim MAX30101 EFD [39] integrated circuit was selected. Maxim electronics has several analog front end integrated circuits (IC) that are suitable for performing PPG. Many of these IC have embedded algorithms for cancelling ambient light, and subroutines to communicate with an external 9 Degree of Freedom (DOF) inertial motion unit (IMU) to cancel motion artifacts. These more sophisticated options had lower sampling rates, less access to raw data, or lacked integrated LED illumination sources and light sensors. The MAX30101 has Red, Green, and IR LED on board to illuminate blood vessels and integrated light sensors at fixed spacings on board, which saves time in optimizing sensor to light spacing, simplifies construction, and provides the basic elements required for reflective PPG. Many libraries exist to communicate with the MAX3010 in Arduino IDE and Micropython. The voltages required to operate the MAX30101 required a specific subsystem to convert between the native 3.3 Volt level of the QTPY and the MAX30101, which communicates over a 1.8 Volt I2C channel and requires a 5 Volt input for its LED driver. A MAX8511 was used to provide a stable 1.8 Volt input to a PCA9306 which translated voltages between the QTPY and MAX30101 to allow communication. A PAM2401 voltage converter was used to boost voltages from the QTPY to the 5 Volt level needed for the LED driver. While a full discussion of the mechanics behind SpO2 is outside the scope of this paper, with the default settings in the firmware, the SpO2 in the MAX30101 has a full scale range of 16,348nA with an LSB resolution of 62.5 pA.

Options for SKT include physically placing a temperature sensor on the skin and using an IR sensor to read temperatures without contact. These methods produce comparable values, especially in the range of temperatures likely to be observed on human skin [40], [41], [42]. While IR measurements display some minor sensitivity to skin color and distance from objects being measured, they have the advantage of not requiring direct skin contact [40], [41], [42]. The Melexis MLX 90614 IR temperature sensor was selected for use in this project. This IC is approved for medical use, is likely to remain in production for the foreseeable future, and has a resolution of 0.02 Celsius [43]. It communicates over I2C with a 3 Volt logic level and has ample libraries available for both Arduino and Micropython environments.

**Fig. 2:**
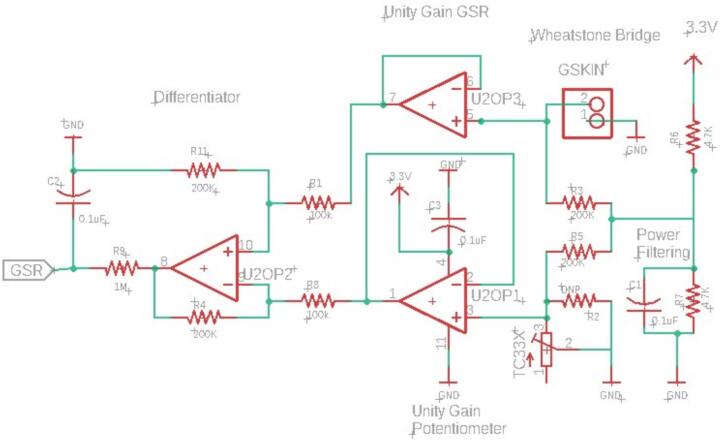
Wheatstone Bridge and Differentiator to measure GSR. The GSKIN port allows for selection of sensors so GSR can be measured from wrist, fingers, or any other location desired.

GSR is a measure of skin conductivity often used in cognitive workload and stress studies [44]. A typical sampling rate for GSR is 1-10 Hz [45]. While there are many ways to measure GSR, one of the simplest takes the form of a Wheatstone Bridge, a pair of Unity Gain Op Amps to boost signal, a final Op Amp differentiator, and an RC filter [46]. The network diagram of this model is shown in Fig. 2. A wide variety of body locations have been proposed for GSR measurements and empirical research has been performed to assess the selection of locations [47]. In our implementation the default location is on the user's wrist to simplify application of the sensors and minimize noise from wire motion artifacts. We have attached this via a plugin port to allow the option of using a wired connection to the fingertips if desired. In keeping with the open-source nature of this project, we have elected to utilize the PyEDA library to perform analysis on GSR responses [20].

For motion tracking, our design incorporates the LSM6DSOX 6 DOF IMU and a LIS3MDL magnetic sensor. The outputs of the 6 DOF IMU and magnetic sensor can be fused to provide 9 DOF linear accelerations and angular velocities in the earths frame of reference. In theory these can allow for tracking of the smoothness of user's motions and be used as a secondary input to correct for motion artifacts observed by the PPG. With the settings coded into the the Cogwatch firmware, the LSM6DSOX measures Acceleration in a range of +/-2g with a Least Significant Bit Resolution (LSB) of 0.61 mg, and measures Angular Velocity in a range of +/-125 degree/s with an LSB of 4.37 degrees/s. The LIS3MDL has a range of +/-4 Gauss with an LSB of 0.14 milli-Gauss. As an initial step, the IMU subsystem communication was verified for the current design. While we hope to use the IMU to evaluate motion patterns as an indicator for stress and cognitive workload in the future, the current application is for a stationary subject. Motion sensors have been included in this stage of the design because: a) Motion is a physiological feature of interest in many studies and adding this in the design stage provides the flexibility to utilize it at a later point and b) If the decision is made to include motion in future studies, the IMU may be useful in identifying sources of noise in other signals and correcting for them through sensor fusion algorithms. The IMU data can be streamed if desired, but it is not used in our current research and the accuracy of the IMU has not been validated against ground truth.

## 4 Design files summary

**Table:** 

Design file name	File type	Open-source license	Location of the file
*CogWatch_main.sch*	Eagle CAD Schematic File	CERN-OHL-S	https://osf.io/szuyw/
*CogWatch_main.brd*	Eagle Cad Board File	CERN-OHL-S	https://osf.io/szuyw/
*CogWatch_sensor.sch*	Eagle CAD Schematic File	CERN-OHL-S	https://osf.io/szuyw/
*CogWatch_sensor.brd*	Eagle Cad Board File	CERN-OHL-S	https://osf.io/szuyw/
*CogWatch_Main.lbr*	Eagle Cad Library File	CERN-OHL-S	https://osf.io/szuyw/
*CaseBase.stl*	3d Printer file	CERN-OHL-S	https://osf.io/szuyw/
*CaseTop.stl*	3d Printer file	CERN-OHL-S	https://osf.io/szuyw/
*UnifiedCase5.SLDPRT*	Solidworks file for case	CERN-OHL-S	https://osf.io/szuyw/
*cogwatch_upload.ino*	Arduino.ino source code	CERN-OHL-S	https://osf.io/szuyw/
*CogWatchBOM.xlsx*	Bill of Materials	CERN-OHL-S	https://osf.io/szuyw/

A bill of materials was generated in an Excel spreadsheet and can be found at: https://osf.io/szuyw/.

## 5 Build instructions

**Fig. 4:**
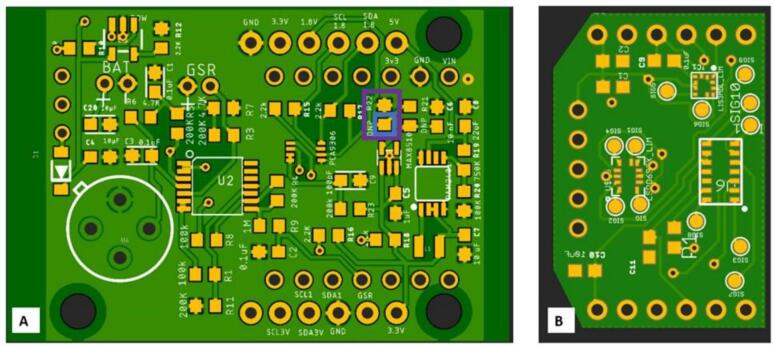
A) CogWatch Main Board. The test point for 1.8 V is shown in the blue box and pads to be joined by solder are shown in the purple box. B) The Sensor Board carries the PPG, 6DOF IMU, and Magnetometer. It allows for the PPG unit to have direct skin contact. The solid gold circles are test points to validate that pads on the IC components have not been bridged.
5) Test round connection point pads on sensor board to verify that no shorts have occurred during reflow. These points can be seen in gold in Fig. 4B. These pads and are connected to ports on the various IC which are note used in this design. They are very close together and can be accidentally bridged. Testing at this stage can save considerable time later on.
a) Optional: If you have access to an oscilloscope, validate that I2C waveforms are seen at the SDA_3V, SCL_3V, SCL_1.8 V, SDA_1.8 V pins. A little bit of time spent doing this will save a lot of time troubleshooting later. A typical output is shown in Fig. 5A. The lines for the 3 V and 1.8 V connections are all shown on same scale and should have identical waveforms when device is on, but data is not being transmitted.

1) Build circuit boards from EagleCad files.
2) Populate circuit boards. The.brd files indicate the location for each of the required electronics components. Each pad should receive a small dab of solder paste and it is possible to complete assembly under a microscope using an Xacto blade and a pair of tweezers. Having a solder screen cut to squeegee solder onto pads and access to a pick and place machine to populate parts simplifies construction significantly.
3) Place board in reflow oven and melt solder according to temperature profile given by manufacturer. Toaster ovens can be used as a low-cost alternative to reflow ovens if needed.
4) Use voltage meter to test voltage outputs at the 5 V, 3 V, and the 1.8 V output pins. Occasionally manufacturers will ship voltage converters that do not work properly. The MAX30101 is very sensitive to overvoltage inputs and difficult to replace if damaged. For the 1.8 V circuit, the pad closest to the MAX8510 voltage converter (R21) should be tested as shown in Fig. 4A. If the voltage is correct solder the pads for R21 together.

**Fig. 5:**
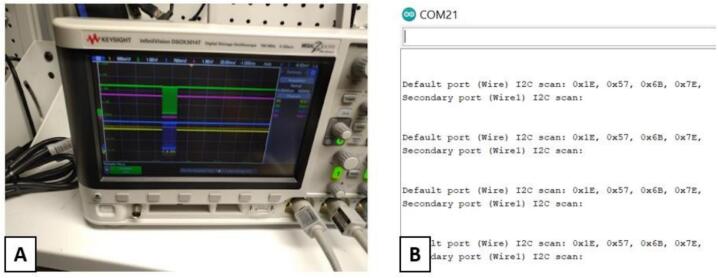
A) Typical output on SCL pins in connected state. The lines for the 3 V and 1.8 V connections are all shown on same scale and should have identical waveforms when device is on, but data is not being transmitted. B) Output from the I2C_Scan app showing expected devices connected.
6) Use pins and solder to attach the mainboard to the sensor board.
b) Optional: Installing the Adafruit testbed library to the Arduino IDE will also allow a simple scan of the system to validate that the ESP32 QT PY can see and connect to all devices. The anticipated output is shown in Fig. 5B.
7) Print top and bottom cases from CaseBase.stl and CaseTop.stl files.
8) Tap the screw holes in bottom case with a 4–40 tap. Put assembled sensor board into bottom case aligned with the window in the bottom case and attach top case with 4–40 screws.

**Fig. 6:**
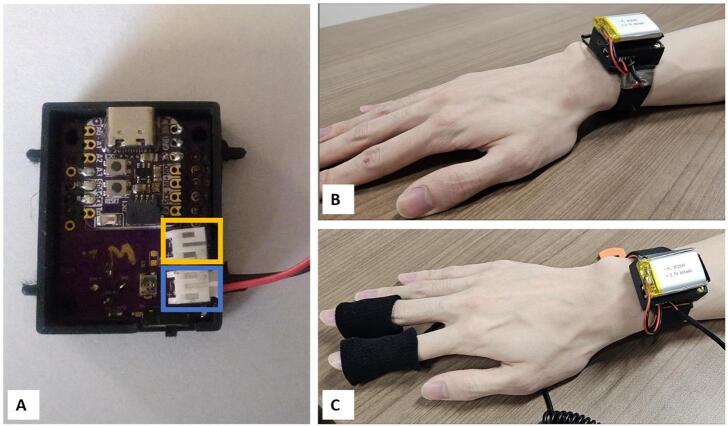
A) The battery port shown circled in Blue and GSR port in Yellow, B) placeholder for wrist mounted GSR sensors, C) placeholder for GSR sensors on fingers.

There are a variety of locations on the body which are viable for placing GSR sensors; popular locations include the fingers and the wrist [47]. While the fingers have slightly higher correlation to stress and cognitive workload, wiring sensors to them does create the potential for noise from wire motion artifacts, and adds the potential for the wires themselves to impede the users’ interactions with the environment. To maximize flexibility in this device we have used a PH2 port to allow wire leads to be plugged in so that electrodes can be placed at the desired location on subjects’ body. Fig. 6 shows which port the GSR sensor and battery should connect to and a sample of two GSR sensor locations. The top one in Fig. 4B shows sensors constructed from conductive tape attached to the wrist location, the figure in Fig. 4C shows remote wired connections to record data from fingertips.

## 6 Operation instructions

### 6.1 Connecting to the CogWatch

**Fig. 3:**
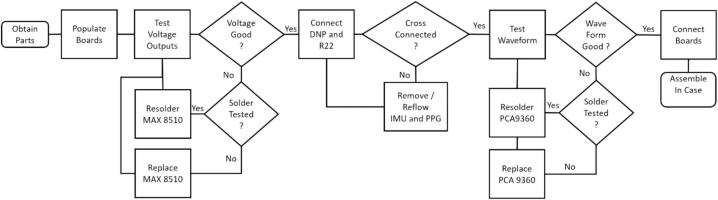
Assembly Flowchart.

These instructions assume that the Cogwatch is being connected to a Windows PC. A flowchart the assembly process can be seen in Fig. 3.

**Fig. 7:**
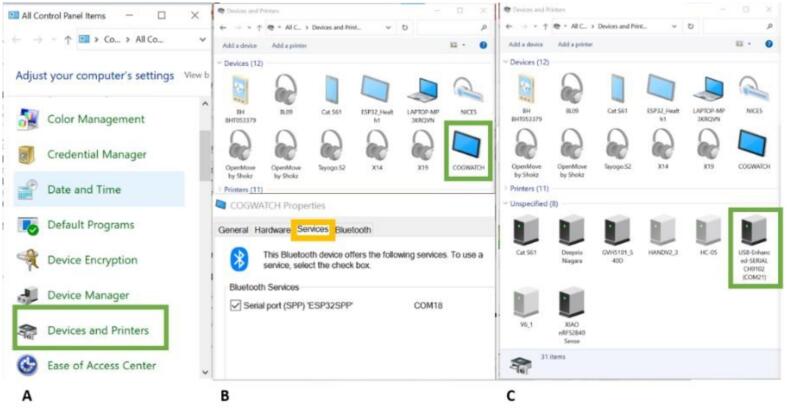
A) Devices and Printers shown in Green Box, B) Bluetooth Device name shown in green box, double click the icon and select the services tab to find the COM port circled in Yellow Box, C) if using standard USB connection then the device will be listed under ‘Unspecified’ and com port will be listed in its name as shown for device in Green Box.
2) To communicate with the CogWatch, a terminal emulator was used. While one is included in the Arduino IDE, it does not allow data logging. The simplest way to log data is to use the YAT terminal emulator and connect to the devices port. The CogWatch can be configured to provide timestamps, or timestamps can be provided by YAT.
  a. To provide timestamps from the device, first update the SSID and Password in the.ino file to match that of the local network. An image of the pertinent lines is shown in Fig. 8A. Once the serial connection is established, send the command ‘T’ over the serial port.

1) Open Windows Control Panel, select Devices and Printers and find the com ports associated with your device as shown in Fig. 7A. If using Bluetooth to connect, the device should appear under devices as in Fig. 7B. If using USB to connect then the device will be listed under ‘Unspecified’ and COM port will be listed in its name as shown for in Fig. 7C.

**Fig. 8:**
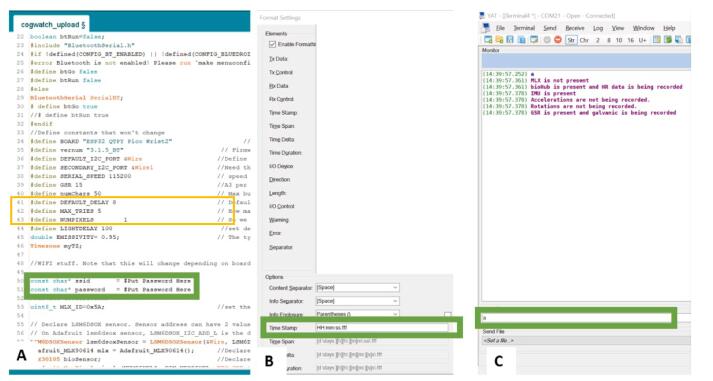
A) Update lines circled in yellow to allow connection to your local WiFi network, B) Insert timestamp as shown in green box, C) the ‘a’ command can be sent to query device to ensure that it is working and give a list of attached sensors.
  b. If using YAT to provide timestamps select View->Show timestamps from the menu bar, and then select View->Format to format the timestamps. In the timestamp box enter HH:mm:ss.fff to set timestamps to hour:minute:second.fraction as seen in Fig. 6B. Then press Cntl-Shift-N to start logging.
3) Once connected to the device there are a few optional commands that can be entered. These commands can be found in the.ino file. The commands most likely to be used include:
  c. A: list current sensors attached to device and their status as seen in Fig. 6C.
  d. P: Lists Sensors being queried by the device.
  e. S: Query all sensors once and for current values.
  f. R: Sets device to run until another command is sent.

## 7 Validation and characterization

### 7.1 Heart rate

**Fig. 9:**
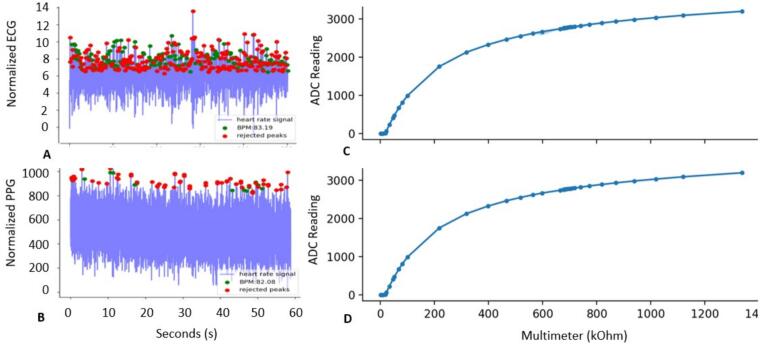
A) shows calculated heart rate based on ECG (Zephyr 3.0 ground truth), B shows PPG (CogWatch) predicted heart rate, Figures C and D show the multimeter measurement of kOhm vs the values registered on the ADC of the ESP32 for selected resistor readings. Fig C highlights min/max in light blue and D shows the standard deviation light blue. It should be noted that both were negligible.

The HeartPy Python library was used to perform HR analysis on both PPG and ECG signals. This library has modules built in to clean noisy data, perform signal processing, and predict RR [24]. Raw data was collected simultaneously using the Cogwatch and a Zephyr Bioharness 3.0 on a single sample user seated in a quiet temperature-controlled office. Local timestamps were used to synchronize the start of recording. HeartPy was used to process the initial data into detectable peaks and calculate heart rate for both ECG and PPG. A comparison of these outputs is shown in Fig. 9A for the normalized ECG and Fig. 9B for the PPG. Analysis of both data collected from the PPG and ECG sensors was performed solely for demonstration purposes and indicated comparable heart rate from both devices. The calculated HR on the two devices differed by a total of 1.3 %.

### 7.2 GSR

As discussed earlier GSR is a measurement of skin conductivity. Conductivity in turn is the inverse of resistance. To validate that the Cogwatch could measure resistance accurately within applicable ranges, a series of resistors varying from 1 kOhm to 620 kOhm were placed in a bread board allowing them to be tested individually and in series. Values from a Fluke 117 Multimeter were collected for each individual resistor and selected combinations along with 1 s of data from the Op Amp Differentiator (OAD). Average values and standard deviations were then calculated from the OAD data at each step. It was found that the minimum recognizable resistance was 18.2 kOhm and that above this resistance a step size of 3.8 kOhm could be distinguished from the background noise. Within the range of 600 kOhm to 1400 kOhm the system displayed near linear behavior as seen in Fig. 9 C and D.

### 7.3 Temperature

IR temperature sensors are commonly used in medical settings. According to manufacturer specifications, the selected IR Temperature sensor (MPL90614-DXX) is approved for use in medical settings. While thermometers are often calibrated in a range of 0C to 100C using ice water and boiling water respectively, this approach would be a far broader range of values than our device needs to operate within. A simpler test is to select an insulated vessel (such as a travel coffee mug) and create a rig for the top opening. The rig is used to mount an IR temperature sensor and position a standard thermocouple to be submerged in the fluid poured into the vessel. Both sensors can then be used to measure the temperature of a fluid poured into the vessel and their measurements compared. Tap water was poured into the glass and the temperature was measured prior to the test using a kitchen thermometer at 27C. Two minutes of data were recorded at a 100 Hz frequency including the IR measured temperature and the thermocouple measured temperature. At the end of the test the temperature was measured again and found to still be approximately 27C.

**Fig. 10:**
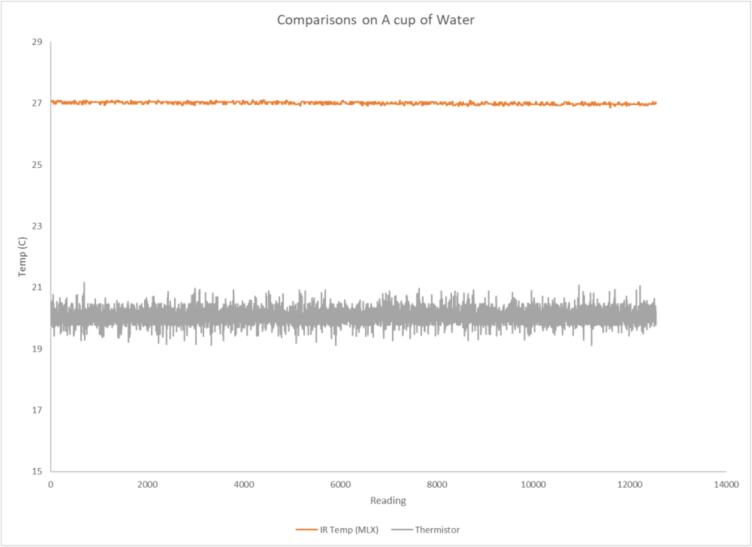
Simultaneous temperature measurements on room temperature water with a thermocouple and the MLX IR temperature sensor. It can be observed that the MLX IR temperature sensor is more consistent in its measurements.

The MPL90614 was found to be both more accurate and display less noise. This is of particular interest because typical stress and cognitive workload applications are more interested in fluctuations of temperature than specific temperatures. As a result, noise is a far more significant concern than accuracy alone. On average the MPL90614 showed a temperature of 27C with an SD of 0.041C and the thermocouple showed a temperature of 20C with an SD of 0.176C. A plot of the variation observed for the two devices can be seen in Fig. 10.

### 7.4 Battery life

The selection of a battery for this design can vary, which will impact the usage. To assess how much energy was required to power the device we attached a 400 milli-Amp Hour (mAh) Lithium-Ion battery to the battery charging system. The battery was charged to full as indicated by the charge light going off, followed by disconnection of the USB cable. A Bluetooth serial connection was established and a command to start streaming data was sent. The time the command was sent and the time the last message from device was received were recorded to establish runtime. This was performed three times and averaged to calculate total run time. The average runtime was 2.47Hours±0.35Hours.

The total run time was then divided by the battery’s energy capacity to estimate how many minutes the Cogwatch could run per mAh of battery.

$$\mathrm{RunTimepermAh}=\frac{\mathrm{tim}e_{\mathrm{Hour}}}{\mathrm{mAh}}*60\frac{m}{\mathrm{hour}}$$

This gave a runtime of $0.37\pm 0.052\frac{\mathrm{minute}}{\mathrm{mAh}}$ which can be used to select appropriately sized batteries.

## 8 Conclusions and future work

A design for the Cogwatch (a low-cost open-source device to monitor physiological indicators) is presented. The sensor systems applicable to our research were validated against existing commercial options. With all sensors running, the device can record at 60 Hz which is adequate for physiological measures. PPG sensors for RR and HR were validated against a Zephyr 3 biosensor on a single seated stationary subject and it was found that the measured heart rates were within 1.3%. The OAD for GSR was compared to a Fluke 117 and it was found that it displayed linear behavior within the range of 600 kOhm to 1400 kOhm with a minimum recognizable step size of 3.8 kOhm. Temperature measurements from the MLX 90614 were compared to a conventional thermocouple with a standard thermometer as ground truth. It was found that while the MLX measured the same temperature as the thermometer, the thermocouple was off by 7 degrees and had significantly higher noise. These results verify that the proposed design can be used to track stress and cognitive workload, which has implications for its use in a variety of applications. Future work includes validating and using the streams of data from the onboard motion sensors to expand applications to experiments involving movement. In addition, the sensors will be used to identify sources of noise in other signals and correcting for them through sensor fusion algorithms. While cognitive workload experimental design was not in the scope of this paper, future work includes utilizing the Cogwatch to distinguish between different levels of workload while performing generalizable cognitive and psychomotor tasks. This includes the extension of prior empirical studies comparing custom built physiological sensors to commercial sensors and subjective cognitive workload metrics across a variety of tasks (perception, physical interaction, memory and decision making) [48], [49].

## Funding

This work was supported by the Naval Engineering Education Consortium [grant number N00174-21-1-0004].

## CRediT authorship contribution statement

**Louis J. Dankovich:** Writing – original draft, Validation, Supervision, Methodology, Conceptualization. **Janell S. Joyner:** Writing – review & editing, Conceptualization. **William He:** Validation, Software. **Ahmad Sesay:** Validation, Software. **Monifa Vaughn-Cooke:** Writing – review & editing, Supervision, Conceptualization, Resources, Project administration, Funding acquisition.

## Declaration of competing interest

The authors declare that they have no known competing financial interests or personal relationships that could have appeared to influence the work reported in this paper.
